# A High Temperature Harvestorer Based on a Photovoltaic Cell and an Oxygen Ion Battery

**DOI:** 10.1021/acsaem.3c02494

**Published:** 2023-12-22

**Authors:** Alexander Schmid, Federico Baiutti, Albert Tarancon, Jürgen Fleig

**Affiliations:** † Institute of Chemical Technologies and Analytics, TU Wien, Getreidemarkt 9, Vienna 1060, Austria; ‡ Catalonia Institute for Energy Research (IREC), Jardins de les Dones de Negre 1, 2a pl, 08930 Sant Adrià del Besòs, Barcelona, Spain; § Catalan Institution for Research and Advanced Studies (ICREA), Passeig Lluís Companys 23, 08010 Barcelona, Spain

**Keywords:** energy harvesting and storage, oxygen ion battery, photovoltaics, solid oxide cell, high temperature photoelectrochemistry

## Abstract

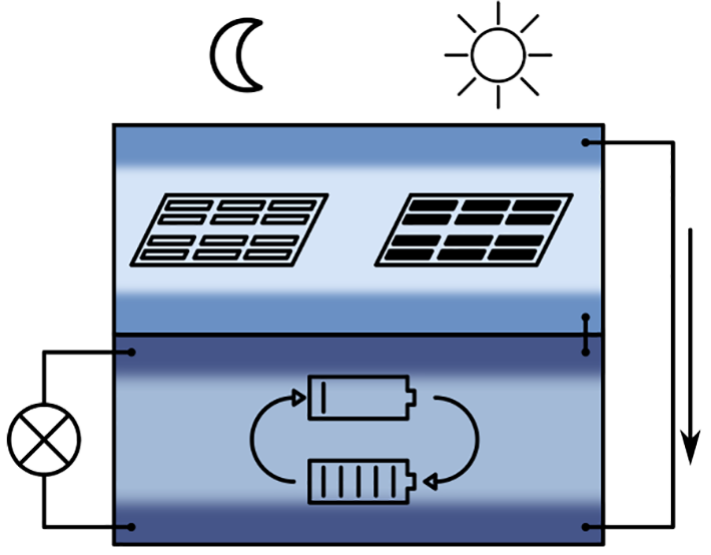

Hybrid devices for combined energy harvesting and storage, i.e., harvestorers, are attractive solutions for powering small autonomous devices (e.g., “smart appliances”, Internet of things nodes), which are ever more prominent as the digitalization and technologization of our society progresses. A concept for a high temperature (HT) harvestorer is presented, and the operational characteristics of a prototype device are discussed. It is based on photovoltaic (PV) energy harvesting and HT electrochemical energy storage. The HT-PV cells employ SrTiO_3_/La_0.9_Sr_0.1_CrO_3−δ_ heterojunctions for energy harvesting and produce photovoltages up to 1 V and photocurrents of several mA cm^−2^ upon UV illumination at 350 °C. Electrochemical energy storage is realized by oxygen ion battery (OIB), a device based on mixed ionic and electronic conducting oxide thin film electrodes and an yttria stabilized zirconia electrolyte. The OIB exhibits capacities of up to 11 mC cm^–2^ (3 μA h cm^−2^) at 0.6 V (350 °C). A prototype harvestorer device was fabricated by integrating an HT-PV and an OIB cell into one device. This harvestorer was operated over several cycles consisting of harvesting and storing energy under illumination, followed by retrieval of the stored energy without illumination. Up to 3.5 mJ cm^–2^ (1 μW h cm^–2^) was stored with energy efficiencies up to 67%. Approaches for further optimization are discussed.

## Introduction

The transition of the energy economy to carbon-neutral, sustainable cycles and the ever-increasing electrification and digitalization of our environment pose demanding challenges to electrical energy technologies. The increasing share of regenerative electricity sources (wind, water, and solar) requires storage systems to ensure a continuous energy supply. Furthermore, the rise of small autonomous devices (“smart appliances”, Internet of things (IoT) nodes) introduces a demand for small, decentralized power generation and storage within the device. Hybrid devices that integrate harvesting of abundant regenerative energy sources and storage of the harvested energy into a single device (harvestorers) are thus highly attractive, especially for small, autonomous appliances such as IoT nodes or smart sensors. Those may be required for a broad range of operation conditions, e.g., temperature, atmosphere, pressure, power, etc. Application in the intermediate temperature range (150 to 400 °C) is of particular relevance for industrial Internet of things solutions, e.g., for powering sensor and actuators for process monitoring. There, commercial energy systems that are designed to operate at room temperatures, such as secondary batteries, are unfavorable because they either pose serious safety hazards requiring further containment measures or simply cannot be operated at high temperatures (lithium ion batteries (LIBs)). In contrast, an all-solid-oxide approach is much safer, as it comprises only nontoxic, nonflammable oxides and is resistant against thermal runaway reactions. In this paper, we present such an all-solid-state oxide approach to a specific harvestorer device operating at elevated temperatures. It is based on energy harvesting via a high temperature (HT)-photovoltaic (PV) cell and electrochemical energy storage via an oxygen ion battery (OIB).

PV cells operating at or close to room temperature are already produced on a large scale, mostly based on silicon, while ceramic oxide solar cells are not yet commercially available and much less commonly investigated.^1^ Room temperature oxide solar cells have been realized based on, e.g., Cu_2_O, ZnO, TiO_2_, or BiFeO_3_.^2−8^ Also, SrTiO_3_ (STO) exhibits a wide range of interactions with light, e.g. photochromism,^9,10^ photoconductivity,^11,12^ and photo-oxidation.^10,13,14^ It forms photoactive heterojunctions with a multitude of materials, for example, Nb:SrTiO_3_/YBa_2_Cu_3_O_7−δ_ (YBCO),^15^ Nb:SrTiO_3_/La_0.5_Ca_0.5_MnO_3−δ_,^16^ or Nb:SrTiO_3_/Cu_2_O.^17^ For our specific high temperature harvestorer it is essential that interfaces between undoped STO single crystals and mixed conductors such as La_0.9_Sr_0.1_CrO_3−δ_ (LSCr) or La_*x*_Sr_1–*x*_MnO_3−δ_ (LSM) generate very high photovoltages of several hundred mV also at temperatures of about 200 to 400 °C.^18,19^

Energy storage at elevated temperature, on the other hand, can be realized by an OIB. This is a novel type of rechargeable battery utilizing mixed ionic electronic conducting (MIEC) oxides for electrochemical energy storage. Such MIEC oxides, e.g., LSM,^20,21^ Ba_*x*_Sr_1–*x*_Co_*y*_Fe_1–*y*_O_3−δ_,^22−24^ La_*x*_Ca_1–*x*_FeO_3−δ_^25,26^ or La_*x*_Sr_1–*x*_Co_*y*_Fe_1–*y*_O_3−δ_,^27−31^ are frequently investigated as electrodes in solid oxide fuel cells or solid oxide electrolysis cells or as oxygen permeation membranes.^32−37^ The MIEC nature of these oxides entails a variability in their oxygen stoichiometry in response to variations in their oxygen chemical potential, which can be modified by an applied voltage. This variation of oxygen stoichiometry by a voltage manifests itself as the chemical capacitance of the oxide^29,38−40^ and can be exploited for electrochemical energy storage, provided that oxygen exchange with the atmosphere is inhibited. The working principle of OIBs is very similar to LIBs, except that here oxide ions are transferred instead of lithium ions, and operation temperatures are thus higher. Unlike LIBs, the materials’ compatibility toward high temperature environments is ensured, as demonstrated by their utilization in state-of-the-art HT energy devices.^35,41,42^ OIB operation at 350 to 400 °C was recently demonstrated for cells with La_0.6_Sr_0.4_FeO_3−δ_ (LSF) thin film cathodes and La_0.5_Sr_0.5_Cr_0.2_Mn_0.8_O_3−δ_ (LSF) anodes.

In this study, we combine these two devices—STO based HT-PV cells and MIEC perovskite based OIB cells—into a single hybrid energy harvesting and storage device, a harvestorer. We first examine the characteristics of the separate HT-PV and OIB cells and thus identify the parameters that are critical for device performance. Based on this, we then demonstrate and characterize operation of a harvestorer proof of concept device: Under illumination, electrical energy generated by the STO HT-PV cell is used to charge the OIB cell, and after removing the illumination, the energy stored in the OIB is retrieved; i.e., the OIB is discharged. Operation over multiple cycles was achieved, and area specific capacities up to 8.3 mC cm^–2^ were repeatedly stored, so far with energy efficiencies up to 67%.

## High Temperature Photovoltaic Generator

An STO based HT-PV cell was used as the harvesting component of the harvestorer device. The basic operating principle of these cells was already described in a previous article.^18^ Here, we briefly summarize the main conclusions before we discuss the specific properties of the HT-PV cell used in the harvestorer device: Heterojunctions between STO single crystals and different ceramic or metallic thin films—LSM, LSCr, Pt, Au—exhibit high photovoltages (up to 1.15 V) when illuminated by ultraviolet light at elevated temperatures (200 to 400 °C). Impedance spectroscopy revealed the presence of a space charge layer at the STO/MIEC oxide or STO/metal interface, which is responsible for the separation of photogenerated electron/hole pairs and thus the creation of a photovoltage. Furthermore, under illumination the photocurrent produced by these cells even increases with time.^18^

**Figure 1:**
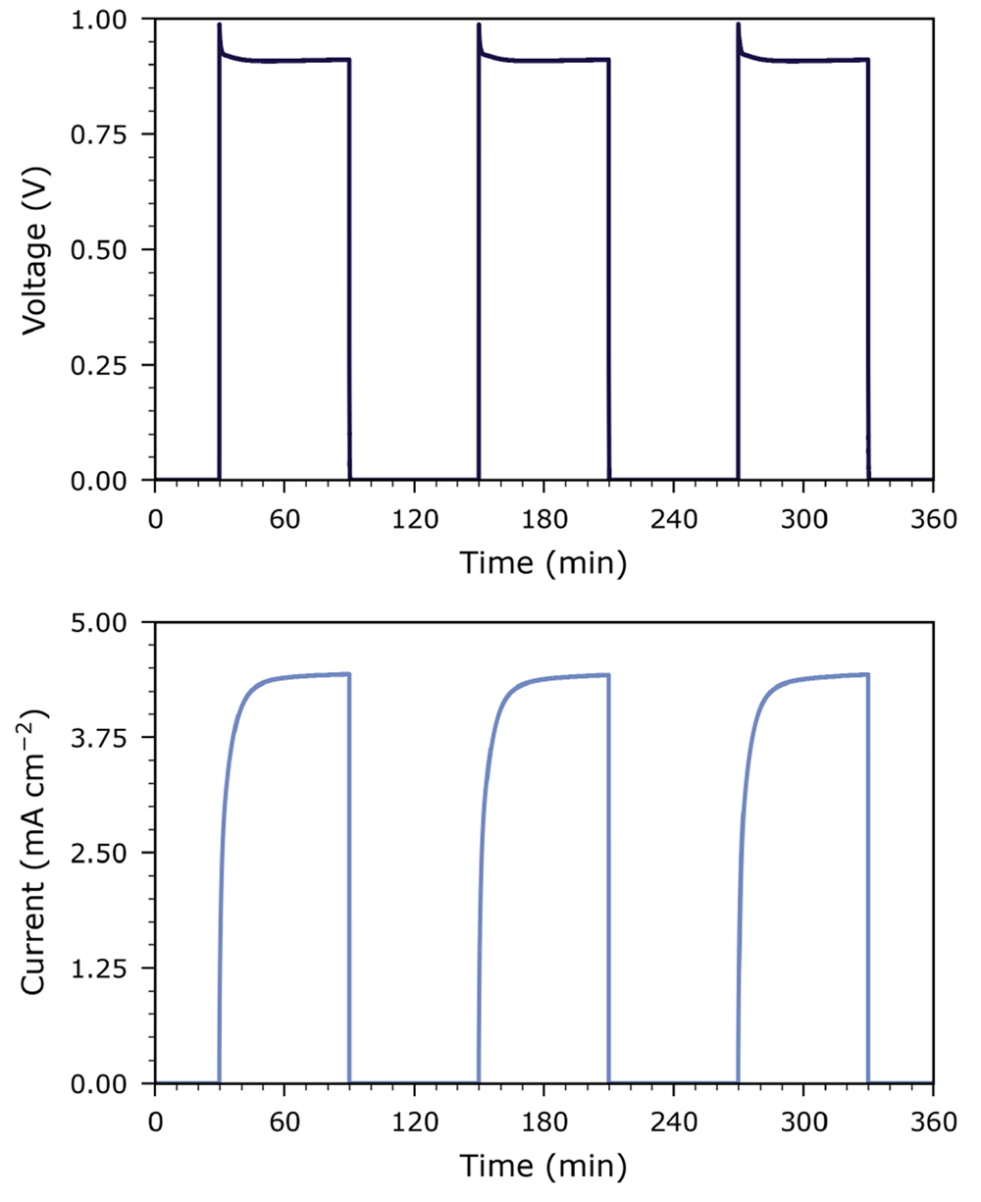
Time evolution of open-circuit photovoltage (a) and short-circuit photocurrent (b) under intermittent UV illumination at 350 °C in a pure O_2_/N_2_ mixture (1 kPa).

In this study, we used HT-PV cells based on LSCr thin film photoelectrodes grown on nominally undoped STO single crystals and YBCO counter electrodes. Figure 1 displays the time evolution of the open-circuit voltage and the short-circuit current of such a cell under intermittent illumination at 350 °C in a pure O_2_/N_2_ mixture ( = 1000 Pa). Upon illumination, the photovoltage rapidly increases to 1 V; this is followed by a slight decrease to 900 mV which then remains stable. The photocurrent, on the other hand, increases more slowly, over a span of about 10 to 20 min and reaches a stable value of 4.4 mA cm^−2^. A detailed, mechanistic analysis of this time dependent behavior and its atmosphere dependence will be presented in a separate article; here, we will only consider experiments performed in synthetic O_2_/N_2_ mixtures after the cell has been illuminated for at least 1 h.

**Figure 2:**
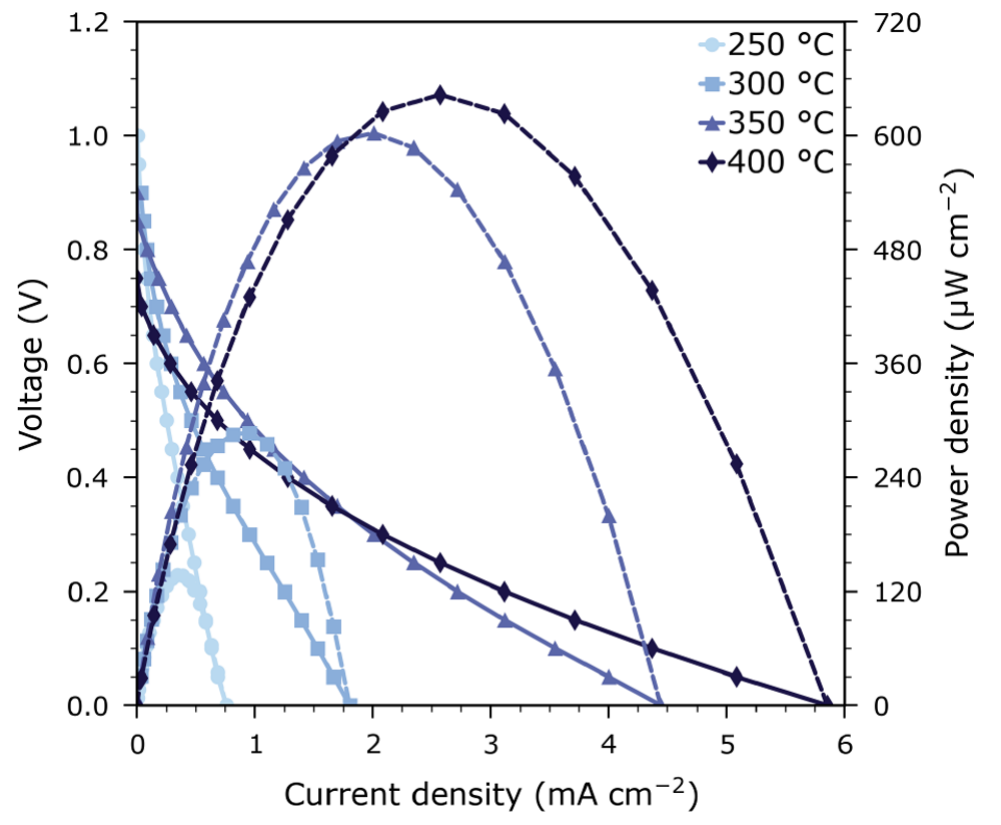
Current–voltage and current–power curves of an STO single crystal based HT-PV cell under UV illumination in a synthetic high purity O_2_/N_2_ atmosphere (1000 Pa) at 250 to 400 °C.

Figure 2 displays the current–voltage and current–power curves of the specific HT-PV cell used in our harvestorer device. Under UV illumination at 350 °C, the cell produced a maximum power of 600 μW cm^–2^ at a current of 2 mA cm^–2^ and a voltage of 300 mV. Notably, the current–voltage relation is distinctly nonlinear. Close to open-circuit conditions, the voltage drops sharply upon increasing the current density, but further increases in current lead to less and less decrease in voltage.

## Oxygen Ion Battery Storage

Oxygen ion batteries, a novel type of electrochemical energy storage device, serve as energy storing components in our combined energy harvesting/storage device. For a detailed description of the operating principle of oxygen ion batteries we refer the reader to a recently published article, demonstrating operation of a proof of concept OIB.^43^ Here, we only briefly repeat the working principle of the OIBs, before we show the characteristics of the specific OIB cell used in this study.

**Figure 3:**
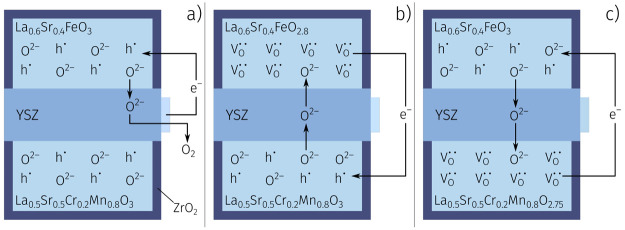
Working principle of an oxygen ion battery: (a) Oxygen is initially pumped out of the cathode into the atmosphere via an auxiliary electrode to precondition the cell. (b) The cell is charged by pumping oxygen from the anode to the cathode. (c) Letting the oxygen flow back discharges the cell. Reprinted from Schmid, A.; Krammer, M.; Fleig, J. Rechargeable Oxide Ion Batteries Based on Mixed Conducting Oxide Electrodes. *Advanced Energy Materials***2023**, *13*, 2203789, DOI: 10.1002/aenm.202203789, under CC-BY license.

OIBs operate by transferring oxygen in the form of oxide ions and electron holes between two MIEC oxide electrodes separated by an oxide ion conducting electrolyte. Figure 3 visualizes the charge and discharge reactions of the OIB: The battery is charged by electrically pumping oxygen from a less reducible anode (e.g., LSCrMn) to an easily reducible cathode (e.g., LSF); see Figure 3b. Vice versa, oxygen flowing back from the cathode to the anode can drive an electronic current through an external load; see Figure 3c. The nominal cell reaction reads
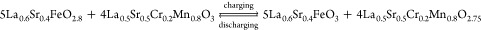
1assuming perfectly matched electrodes and complete charging and discharging. Overall, the operation principle is very similar to that of LIBs, except that here oxygen is used as ionic charge carrier instead of lithium, and thus, higher operation temperatures (300 to 500 °C) are needed.

However, OIBs exhibit one particularity not found in other battery types. By using a third auxiliary electrode exposed to the surrounding atmosphere, oxygen can be added to or removed from the storage electrodes of the OIB cell. In this way, oxygen lost to parasitic leakage can be replaced, and lost cell capacity can be repeatedly recovered; see Figure 3a. More specifically, oxygen may leak in from the atmosphere, and thus, oxygen needs to be pumped out of the cell to recover cell capacity lost to this leakage. This recovery step is also required before first operation of the cell, as both electrodes are fully oxidized (i.e., filled with oxygen) after fabrication, and thus, one electrode needs to be reduced first.

**Figure 4:**
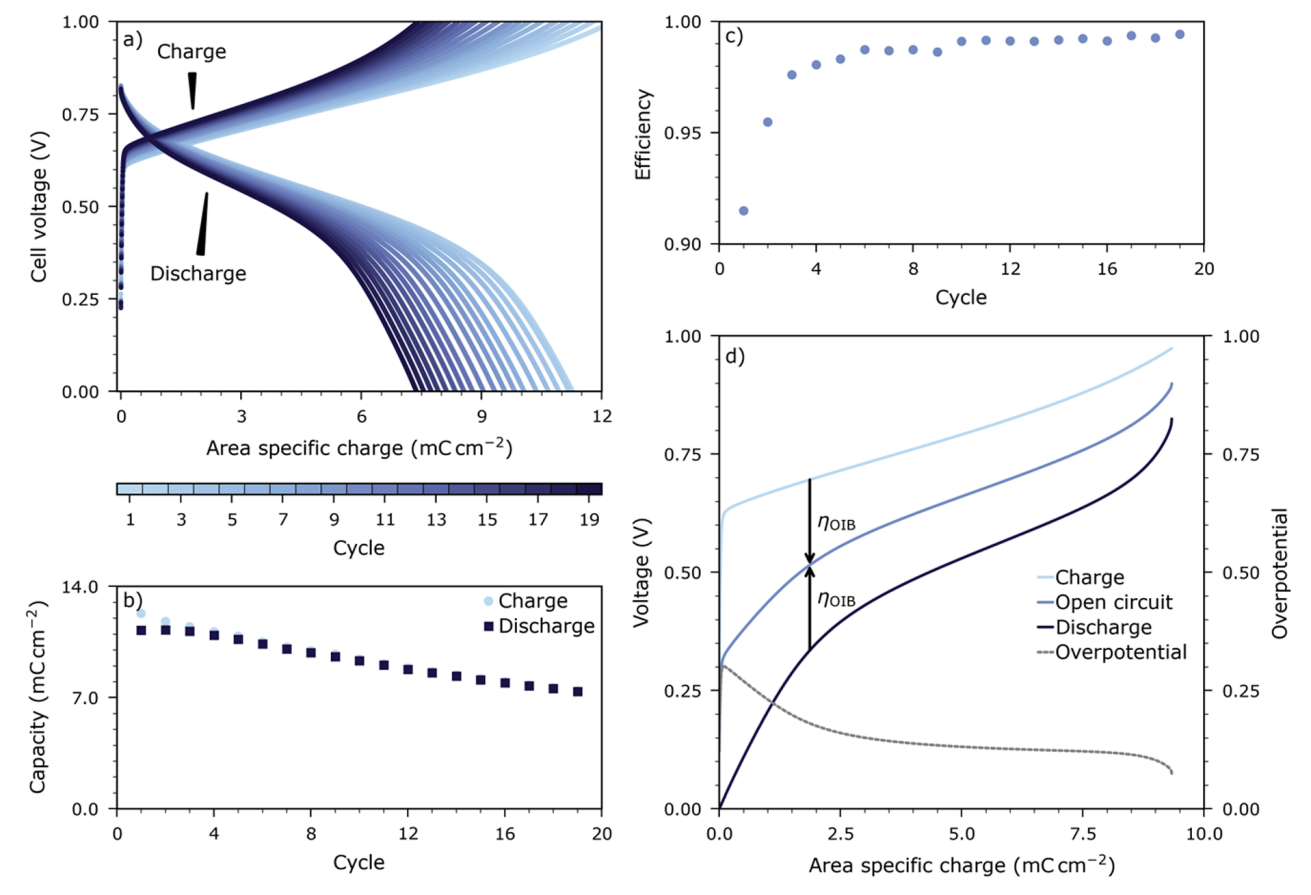
(a) Charge–voltage curves of OIB cell with LSF cathode and LSCrMn anode, measured at 350 °C in 10 mbar O_2_ with a current of 17 μA cm^–2^ between 0 and 1 V. Nineteen cycles are shown; the initial charging step is excluded. Capacities are normalized to the average area of both electrodes. (b) Electrode capacity extracted from data in (a). (c) Corresponding coulomb efficiency. (d) Cell voltage during one charge–discharge cycle together with the corresponding open-circuit voltage and overpotential. The overpotential was estimated as half the difference between charge and discharge voltage at the same state of charge.

In our specific harvestorer device, the OIB cell consists of an LSF thin film cathode and an LSCrMn anode, both grown by pulsed laser deposition on an yttria stabilized zirconia (YSZ) single crystal electrolyte. On top of both electrodes, a dense zirconia layer was deposited to minimize oxygen exchange with the atmosphere. Figure 4a shows the charge–discharge characteristics of this OIB cell over multiple cycles. The area specific cell capacity initially starts at 11.3 mC cm^–2^ and continually decreases by about 2% per cycle; see Figure 4b. This is also reflected by a coulomb efficiency below unity; see Figure 4c. Figure 4d shows one charge–discharge cycle together with the corresponding open-circuit voltage and overpotentials—a discussion of these overpotentials is given below.

**Figure 5:**
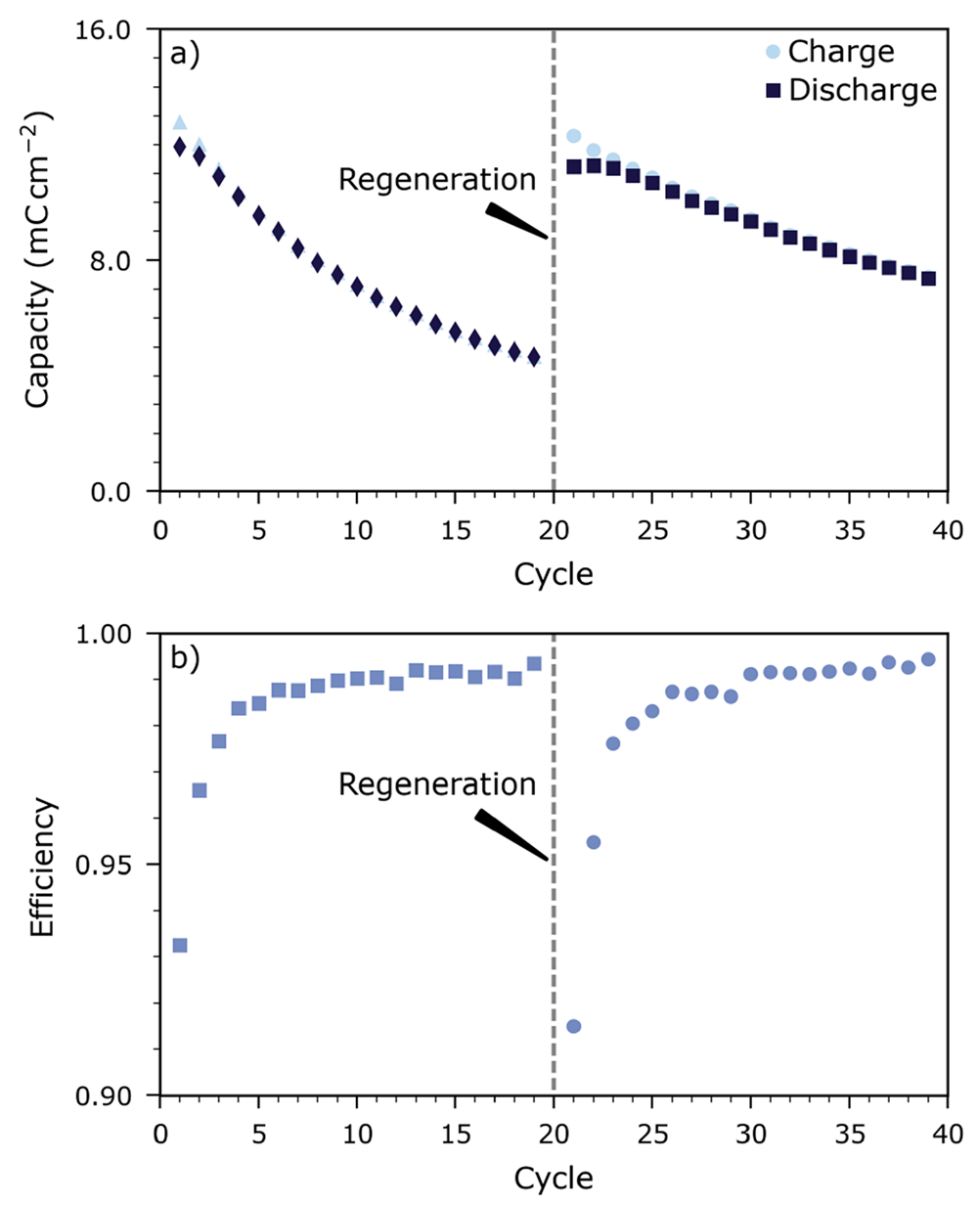
Capacities (a) and coulomb efficiencies (b) recorded over 19 cycles before and after a regeneration step, i.e., after removal of leaked oxygen from the LSF cathode via the auxiliary electrode.

The decrease in cell capacity and the below unity coulomb efficiency are caused by imperfections in the sealing layer, allowing small quantities of oxygen from the atmosphere to leak into the storage electrodes. This oxygen leakage causes a filling of oxygen vacancies in the electrodes and thus a reduced electrode capacity. Over the first few cycles, the efficiency increases from 95% to 99%. The exact origin of this increase is not yet clear but likely also is a consequence of a shift of both electrodes to a more oxidized state due to oxygen leaking in through the imperfect sealing. However, as discussed above, the capacity loss is reversible: By removing the leaked oxygen from the LSF cathode via the auxiliary electrode, the lost capacity can be recovered repeatedly, as shown in Figure 5.

The overpotential of the OIB is constant over most of its charge–discharge cycle, except close to the fully discharged state where the overpotentials are very high, in the range of several hundred mV. Close to the fully discharged state the LSCrMn anode is fully oxidized; i.e., it contains virtually no oxygen vacancies, and thus, its ionic transport resistance contributes significantly to the total cell resistance. However, once the cell gets charged, oxygen vacancies are formed in the anode, and its transport resistance becomes much lower. The total cell resistance is then predominantly caused by the ionic transport resistance of the thick YSZ electrolyte and contributions from transport across the interfaces between the electrodes and electrolyte.^43^ At 17 μA cm^–2^, the average overpotential loss of the OIB cell is 95 mV, corresponding to an internal cell resistance of 5.6 kΩ cm^2^.

## Harvestorer Device

**Figure 6:**
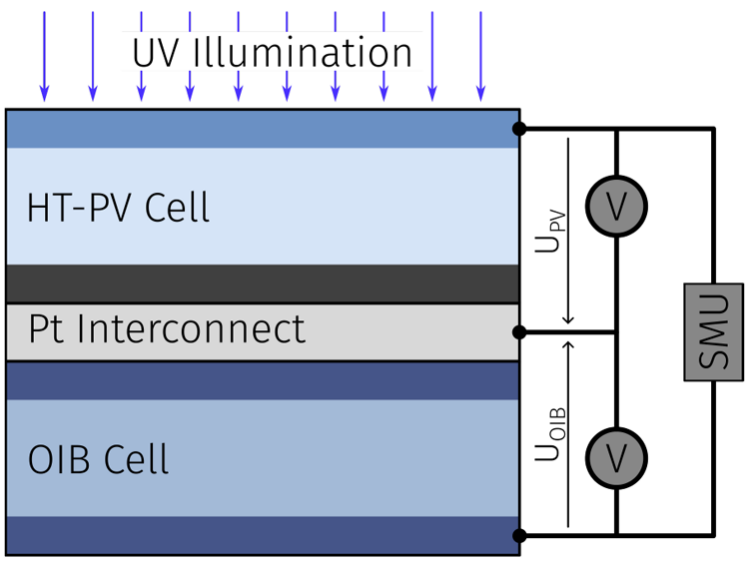
Schematic stack-up of the harvestorer cell, created by connecting the HT-PV and OIB cells with a Pt interconnect including external connections used for photoelectrochemical characterization.

**Figure 7:**
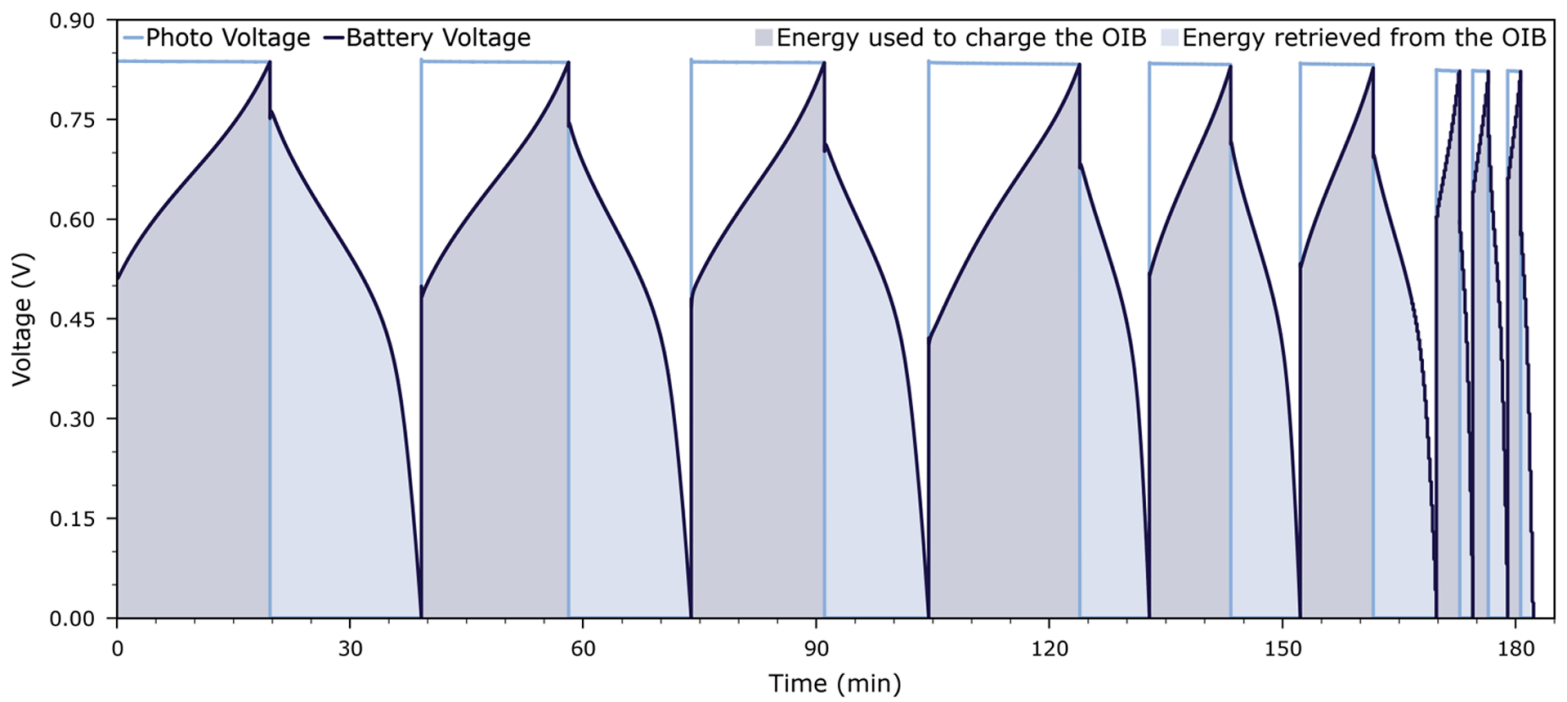
Time evolution of photovoltage and battery voltage in a harvestorer cell, recorded over 9 consecutive charge–discharge cycles. The OIB cell was charged and discharged with constant currents of 5 μA (cycles 1–3), 10 μA (cycles 4–6), and 20 μA (cycles 7–9) at 350 °C in 1 kPa O_2_.

A combined energy harvesting and storage device (harvestorer) was fabricated by combining a HT-PV cell and a OIB cell; see Figure 6. Details of the different layers and their preparation are given in the Experimental Section. This harvestorer cell was then illuminated, and the photocurrent produced by the HT-PV cell component was used to photocharge the OIB component. Then, the illumination was removed, and the OIB was discharged. Figure 7 displays a time series consisting of 9 charge–discharge segments with 3 different charging rates.

**Figure 8:**
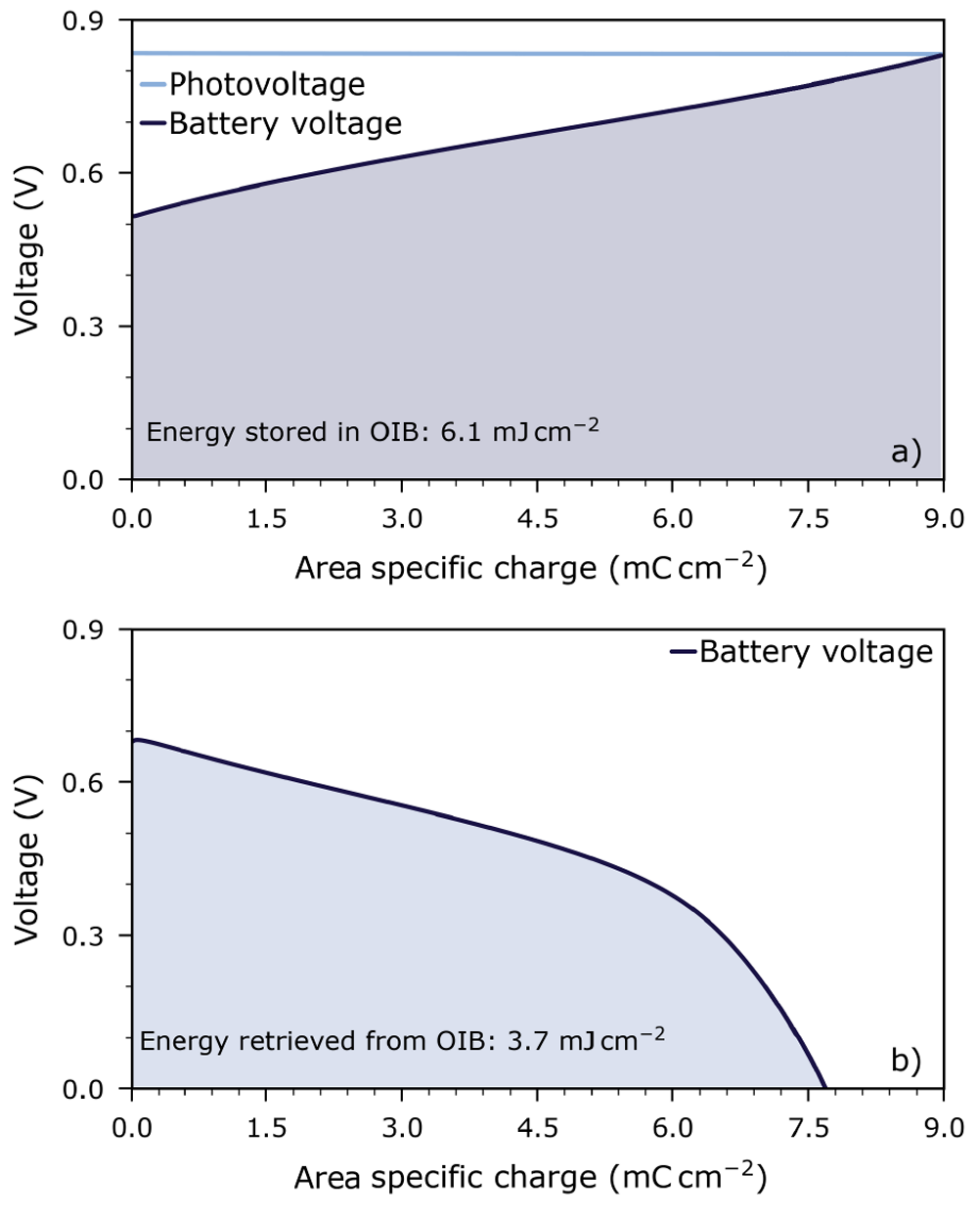
Charge–voltage characteristics of the OIB cell during charging under illumination (a) and during discharging without illumination (b). Data were recorded using a constant current density of 17 μA cm^–2^ at 350 °C in 1000 Pa O_2_.

From these data, we can calculate the charge and energy used to charge the OIB and then retrieved from the OIB, respectively. By integration, we find
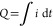
2and
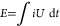
3where *i* is the current density, *U* is the battery voltage, *Q* and *E* are (area specific) charge and energy, respectively, and *t* is time. Figure 8 shows an exemplary charge–voltage characteristic extracted from one photocharge segment under illumination and the following discharge segment, both recorded using a current of 10 μA (the fifth cycle in Figure 7). This current corresponds to current densities of 13 μA cm^–2^ in the HT-PV cell, and 17 μA cm^–2^ in the OIB cell, respectively.

A charge of 9 mC cm^–2^ was transferred to the OIB during photocharging; see Figure 8a. During discharging, 7.7 mC cm^–2^ was retrieved from the OIB cell; this corresponds to a coulomb efficiency of 86%. As discussed above, imperfections in the oxygen blocking sealing layer lead to oxygen leaking from the atmosphere into the device and thus cause a self-discharge of the OIB. The theoretical capacity of the OIB, derived from defect chemical considerations,^43^ is much larger (approximately 36 mC cm^–2^), and only 25% of the theoretical OIB storage capacity is utilized. The usable OIB cell capacity is limited by the specific photovoltage under load (0.83 V) produced by the HT-PV cell, which determines the maximum battery voltage during photocharging (including overpotentials). Utilizing the entire OIB storage capacity would thus require higher photovoltages or lower losses (overpotentials) at internal resistances. The energy used to charge the OIB was 6 mJ cm^–2^, of which 3.6 mJ cm^–2^ was recovered during discharge, resulting in an energy efficiency of 61%. Alternatively, one could charge the OIB without imposing a fixed current, i.e., letting the internal resistances limit the current. This would lead to additional energy dissipation at the internal resistance (mainly of the thick OIB electrolyte) and thus reduce the energy storage efficiency to about 55%. Table 1 lists the performance characteristics of the harvester device under different operating conditions.

**Table 1:** Operation Characteristics of the Harvestorer Device: Charge and Energy Densities Used to Charge the OIB Cell under Illumination (*Q*_C_, *E*_C_) and Retrieved from the OIB Cell without Illumination (*Q*_D_, *E*_D_), Together with the Corresponding Charge and Energy Related Efficiencies (ω_C_, ω_E_) for Different Temperatures (*T*), Oxygen Partial Pressures  and Currents (*I*)^a^

			(mC cm^–2^)			(mJ cm^–2^)			(mV)	
*T* (°C)	*p*O_2_ (Pa)	*I* (μA)	*Q*_C_	*Q*_D_	ω_C_ (%)	*E*_C_	*E*_D_	ω_E_ (%)	η_PV_	η_OIB_
350	25	10	14.7	8.4	60	9.1	3.5	40	112	105
	1000	5	8.0	7.0	87	5.3	3.5	67	64	78
		10	11.2	7.5	73	7.3	3.6	53	67	90
		20	3.5	3.1	90	2.5	1.2	49	77	169
400	25	5	4.4	3.6	84	2.6	1.6	62	44	78
		10	6.8	3.6	67	3.9	1.5	47	49	100
		20	3.5	3.1	90	2.1	1.3	59	56	108
		50	1.6	1.2	76	1.0	0.3	30	75	219

a Also listed are the overpotentials (η_PV_, η_OIB_) originating from the HT-PV and OIB cells, both averaged over an entire cycle. Each datum is an average of three consecutively recorded photocharge–discharge cycles.

**Figure 9:**
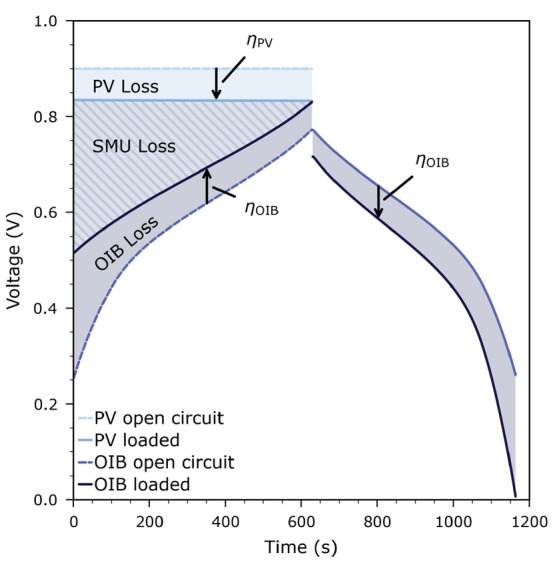
Time evolution of the open-circuit and loaded photovoltage and battery voltage during one photocharge–discharge cycle at 350 °C in 1000 Pa O_2_ using a constant current of 10 μA.

To understand the limiting factors for the harvestorer device performance and assess the most promising approach for optimizing said performance, we now analyze the individual loss processes and the corresponding overpotentials in detail. We again use the cycle recorded at 350 °C with a current of 10 μA (i.e., the same as depicted in Figure 8) as the basis for our discussion. Figure 9 displays the time evolution of the photovoltage and battery voltage, both under load and under open-circuit conditions. Under open-circuit conditions, the HT-PV cell produces a photovoltage of 900 mV. Under load, this voltage decreases to 830 mV, i.e., η_PV_ = 70 mV. Open-circuit voltage and photovoltage under load were both determined separately, see Figure 2, and the loaded photovoltage was also measured continuously during photocharging. This photovoltage under load is now available to charge the OIB cell. Due to the experiment being performed with a constant current, part of this voltage is dissipated in the control circuitry of the external source meter unit (SMU), and the remaining voltage is applied to the OIB cell.

At the beginning of the charging phase, 315 mV is dissipated by the SMU and the remaining 515 mV is applied to the OIB cell. During the charging phase, this battery voltage under load increases until it reaches the photovoltage under load (830 mV), and correspondingly, the voltage dissipated by the SMU decreases to zero. Part of the voltage applied to the OIB drops across its internal resistance, and thus, the open-circuit voltage of the OIB cell only reaches 775 mV, i.e., η_OIB_ = 55 mV. During discharging of the OIB, the same overpotential reduces the OIB cell’s open-circuit voltage and thus the voltage of the OIB cell under load starts at 720 mV. Please note: The OIB overpotentials given here refer to the fully charged state, i.e., to the switch from photocharging to discharging of the OIB. However, in contrast to η_PV_, the value of η_OIB_ changes during charging/discharging. Close to the fully discharged state, η_OIB_ is higher, resulting in OIB overpotential η_OIB_ of 95 mV averaged over the entire specific cycle, and an average value of 90 mV over three cycles.

From this analysis, it becomes clear that the overpotential losses at both harvestorer components are very similar and thus that device performance is equally limited by both component’s internal resistances. This, however, is true only for the rather low current densities used in this study. Once we aim at higher power densities (and thus at higher current densities), we have to consider that the overpotential losses in the OIB cell increase more or less linearly with the current, corresponding to the predominantly ohmic nature of its ionic transport resistance. In contrast, the current–voltage characteristic of the HT-PV cell is much closer to an exponential relation, as discussed above, and thus, the overpotential losses increase much less with increasing current.

**Figure 10:**
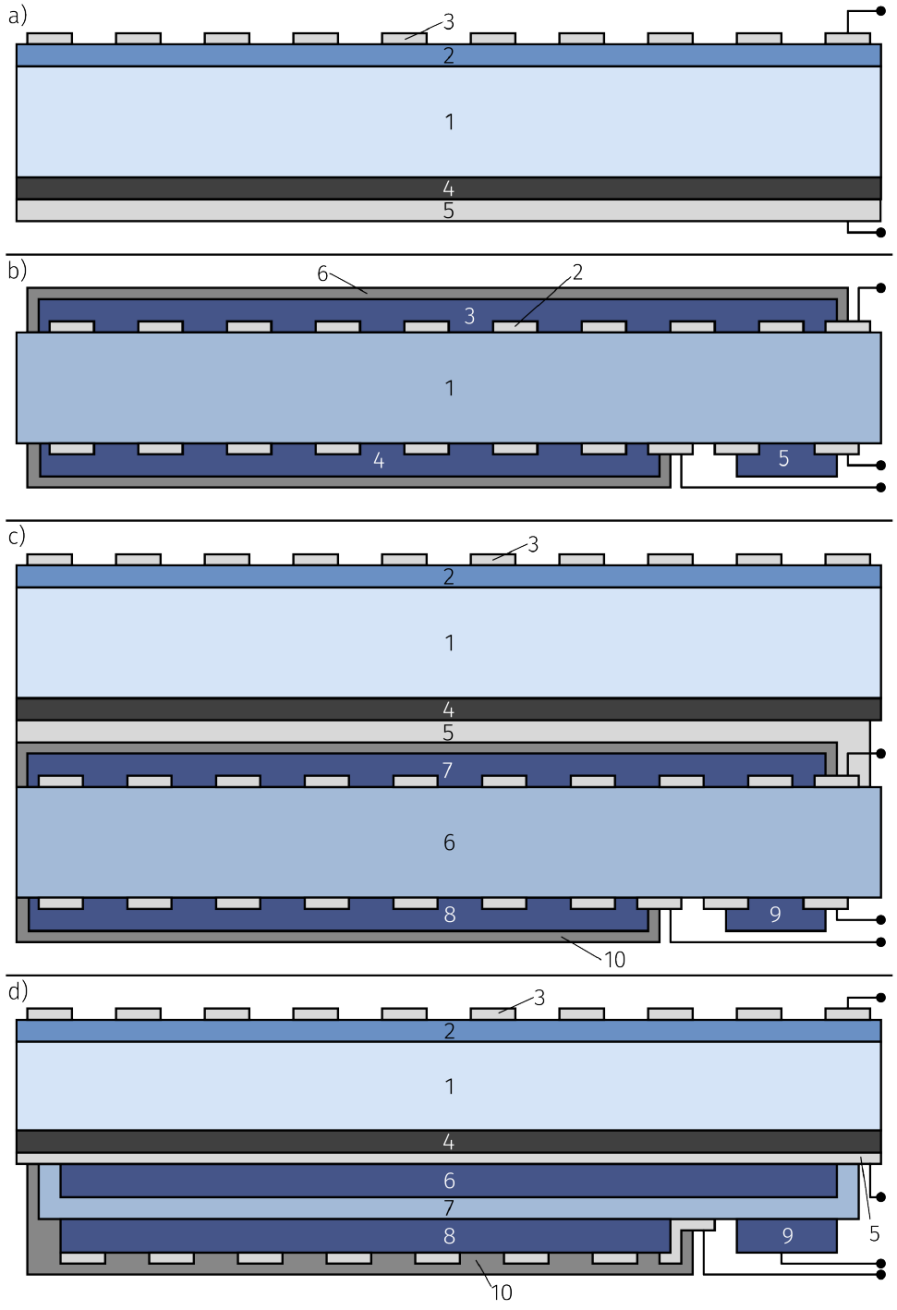
(a) HT-PV cell consisting of STO single crystal substrate (1), LSCr photo-electrode (2), Pt current collector—15 μm strip width and 35 μm mesh width (3), dense YBCO counter electrode (4) and porous Pt contacting layer (5). (b) OIB consisting of YSZ single crystal substrate (1), Pt current collector (2), LSF cathode (3), LSCrMn anode (4), LSCrMn auxiliary electrode (5) and oxygen blocking ZrO_2_ layer (6). (c) harvestorer device consisting of STO single crystal substrate (1), LSCr photo-electrode (2), Pt current collector (3), dense YBCO counter electrode (4), porous Pt connecting electrode (5), YSZ single crystal substrate (6), LSF cathode (7), LSCrMn anode (8), LSCrMn auxiliary electrode (9) and oxygen blocking ZrO_2_ layer (10). (d) Proposed design for an STO supported monolithic harvestorer cell consisting of STO single crystal substrate (1), LSCr photo-electrode (2), Pt current collector (3), YBCO counter electrode (4), dense Pt layer (5), LSF cathode (6), YSZ electrolyte film (7), LSCrMn anode (8), LSCrMn auxiliary electrode (9) and oxygen blocking ZrO_2_ layer (10).

Thus, if we aim at current densities of, for example, 1 mA cm^−2^ (an increase by a factor of 60), our HT-PV cell would still produce a photovoltage of 480 mV (η_PV_ = 420 mV). At such currents, our single crystal OIB cell, however, would require several volts to be charged. Clearly, this is much greater than the voltage provided by the HT-PV cell. Thus, current densities in the mA range require the use of OIB cells with a drastically lower internal resistance. In our current OIB cells, the major source of internal losses is the transport resistance through the thick electrolyte, and thus, replacing the thick single crystal by a thin film electrolyte is a promising approach to minimizing OIB internal losses. For example, we can estimate that a 1 to 5 μm thick film of YSZ, gadolinia-doped ceria, or samaria-doped ceria will lead to internal resistances in the range of 5 to 50 Ω cm^2^ at 350 °C and thus make operation in the mA cm^−2^ range feasible. Alternatively, this approach would allow lower operation temperatures, e.g., of 200 °C, with currents in the 10 μA cm^–2^ range. The HT-PV cell, on the other hand, would benefit much less from replacing the STO single crystal by a thin film as the internal resistance of the HT-PV cell is dominated by the space charge resistance. In contrast, the electron transport through the STO bulk only constitutes 1 to 5% of the internal losses. Therefore, a harvestorer consisting of a thin film electrolyte OIB grown on an STO single crystal HT-PV generator, is highly attractive; a possible device structure is shown in Figure 10d.

## Conclusions

High temperature (HT)-photovoltaic (PV) cells based on heterojunctions between SrTiO_3_ (STO) single crystals and La_0.9_Sr_0.1_CrO_3−δ_ (LSCr) thin film photoelectrodes were fabricated and characterized. They exhibited open-circuit voltages of 900 mV, short-circuit currents of 4.5 mA cm^–2^, and maximum power densities of 600 μW cm^–2^ at 350 °C. Oxygen ion batteries (OIBs) based on mixed ionic and electronic conducting (MIEC) oxide thin films were prepared on yttria stabilized zirconia (YSZ) single crystal electrolyte substrates. These showed capacities between 7 mC cm^–2^ and 11 mC cm^–2^ at 350 °C. Capacity losses caused by imperfect sealing against the atmosphere could be repeatedly regenerated via an auxiliary electrode exposed to the atmosphere. The overpotential losses during charging and discharging of the OIB are dominated by the thick single crystal electrolyte and the corresponding ionic transport resistance.

HT-PV and OIB cells were combined to create an integrated all-solid-state energy harvesting and storage device, a harvestorer. These harvestorers were charged by illuminating them with UV light: The energy was generated by the PV cells and stored chemically in the OIB cell. Up to 8.3 mC cm^–2^ (3.6 mJ cm^–2^) could be stored and retrieved, with a coulomb efficiency of 60 to 90% (30 to 67% with respect to energy). Overpotentials due to the transport resistance of the thick YSZ single crystal electrolyte were again responsible for a large part of the losses.

Overall, results show that harvestorers based on STO based solar cells and MIEC oxide based OIBs are viable solutions for single device power harvesting and storage. So far, the major limitation for device performance is the thick YSZ single crystal, and thus, significant performance increases are expected when replacing the YSZ crystal by a thin film electrolyte. We estimate that such thin film electrolytes will make current densities in the mA cm^−2^ range viable for OIBs and thus bring OIBs into the current range possible for the STO based PV cells. Further optimization toward decreasing the operational temperature as well as allowing the PV cell operation under visible light will greatly extend the possibility to apply the developed harvestorer in real environments for powering small devices, e.g., for industrial Internet of things.

## Experimental Section

### Sample Preparation

STO single crystals (10 mm × 10 mm × 0.5 mm, (100) orientation, polished on one flat side) were used as substrates for preparation of HT-PV cells. Prior to thin film deposition, the crystals were cleaned in an ultrasonic bath in Extran, water, and ethanol and afterward annealed at 900 °C for 1 h in 4 Pa O_2_ inside the pulsed laser deposition (PLD) chamber. YBa_2_Cu_3_O_7−δ_ (YBCO) counter electrodes (50 nm) were grown by PLD on the unpolished flat side of the crystals, using the parameters in Table 2. LSCr thin films (100 nm thickness) were grown on the polished side, also by PLD. Pt current collector grids (100 nm thickness, 35 μm mesh width, 15 μm strip width) were deposited on top of the LSCr films by lift-off photo lithography and DC magnetron sputtering. Pt paste was brushed on top of the YBCO counter electrodes for better electrical contacting. Figure 10a shows a sketch of the resulting structure.

**Table 2:** Deposition Parameters Used for PLD

material	substrate temperature (°C)	oxygen pressure (Pa)	growth rate (nm/pulse)
LSCr	700	1.5	16
LSF	600	4.0	14
LSCrMn	700	1.5	18
ZrO_2_	650	1.5	13
YBCO	800	4.0	15

OIB cells were prepared on YSZ single crystals (10 mm × 10 mm × 0.5 mm, (100) orientation, polished on both flat sides). Crystals were cleaned as described above and annealed in air at 1200 °C for 2 h prior to thin film deposition. Pt current collector grids were prepared on both sides as described above except that an additional 5 nm Ti layer was deposited between Pt and oxide to improve adhesion. MIEC electrode thin films (369 nm La_0.6_Sr_0.4_FeO_3−δ_ (LSF), 333 nm La_0.5_Sr_0.5_Cr_0.2_Mn_0.8_O_3−δ_ (LSCrMn)) were deposited on top of the current collector grids by PLD using the parameters in Table 2. Finally, a dense zirconia layer (1.2 μm) was deposited by PLD on top of the MIEC working and counter electrodes to isolate them from the atmosphere. Undoped zirconia was chosen as it has low ionic and electronic conductivity as well as good chemical compatibility for the perovskite electrodes. A laser fluence of 1.1 J cm^–2^, a pulse energy of 110 mJ, and a pulse repetition frequency of 10 Hz were employed for all depositions except YBCO, where a pulse energy of 140 mJ and a fluence of 1.4 J cm^–2^ were used. Alumina shadow masks were used during PLD and sputtering to macroscopically structure the films. Figure 10b shows a sketch of the resulting sample design. A combined power generator and storage sample (harvestorer) was prepared by stacking the HT-PV cell and the OIB cell; see Figure 10c.

Deposition targets for PLD were produced from powders by uniaxial pressing (71 MPa) and sintering in air for 12 h at 900 °C (YBCO target), 1200 °C (other MIEC targets), and 1600 °C (ZrO_2_ target), respectively. ZrO_2_ and YBCO powders were bought from Sigma; other MIEC oxide powders were prepared via Pechini’s route: Metal precursors (Cr(NO_3_)_3_, Mn(CO_3_)_2_, Fe, La_2_O_3_, SrCO_3_, purity >99.995%) were dissolved in dilute nitric acid, and citric acid was added in a molar ratio of 1:1 with respect to total cations. The solution was stirred at 90 °C for two hours and afterward evaporated until completely dry. The dry foam was then further heated until self-ignition and combustion occurred and subsequently calcined in air at 850 °C for 2 h.

### Photoelectrochemical Characterization

**Figure 11:**
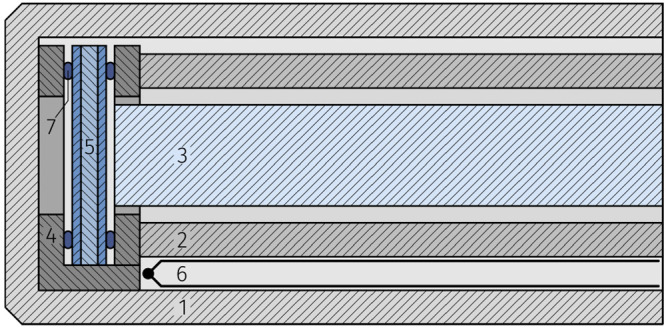
Measurement stage used for photoelectrochemical characterization, consisting of outer silica tube (1), inner silica tube (2), silica light guide (3), alumina sample holder (4), sample (5), thermocouple (6) and electrical contacting (7).

Samples were mounted and electrically contacted in an alumina sample holder enclosed in a tube of fused silica and placed inside a tube furnace; see Figure 11. Samples were illuminated by UV light via a fused silica rod (1 cm diameter) serving as light guide, placed through a circular cut-out in the sample holder. An LED lamp (LZ4 LuxiGen UV LED Emitter, LED Engin, USA) with a nominal power of 2.9 W and a wavelength of 365 nm was used as the UV light source, resulting in a UV power of 55 mW (70 mW cm^–2^) at the sample. Due to the heat imparted on the sample by the UV radiation, sample temperatures under illumination were slightly higher than in the dark, despite identical furnace temperatures. The nominal temperatures given in this study refer to the temperatures under illumination, whereas the corresponding temperatures in the dark were about 2 to 5 °C lower.

DC measurements were performed using a Keithley 2000 digital multimeter and a Keithley 2600 SMU. All photovoltages and currents in this study are given relative to the LSCr working electrode; i.e., a positive photovoltage means that the counter electrode is at a more positive potential than the LSCr working electrode. Likewise, a positive photocurrent means that an external current flows from the counter electrode to the working electrode. OIB voltages are given relative to the LSCrMn anode, i.e. a positive battery voltage means that the LSF cathode is at a more positive potential than the LSCrMn anode. Current and power of the HT-PV cells were normalized to the illuminated area including the area shaded by the current collector, i.e., to a circle of 1 cm diameter (0.79 cm^2^). Current and power of the OIB measurements were normalized to the working electrode area, i.e., to 0.59 cm^2^.
